# Correction: Parental involvement in school-based mental health interventions for young people in low-resource settings: A qualitative study from Zimbabwe and Ghana

**DOI:** 10.1371/journal.pone.0334227

**Published:** 2025-10-09

**Authors:** Rufaro Hamish Mushonga, Rebecca Jopling, Franklin Glozah, Tiny Kamvura, Suzanne Dodd, Denford Gudyanga, Arnold Maramba, Edith Dambayi, Christopher Abio Ayuure, Tarisai Bere, Fabian Sebastian Achana, Lucy Owusu, Dixon Chibanda, Melanie Abas, Benedict Weobong, Moses Kumwenda

There are errors in the author affiliations. The correct affiliations are as follows:

Rufaro Hamish Mushonga^1,2^, Rebecca Jopling^1^, Franklin Glozah^3^, Tiny Kamvura^4^, Suzanne Dodd^1^, Denford Gudyanga^1^, Arnold Maramba^4^, Edith Dambayi^5^, Christopher Abio Ayuure^6^, Tarisai Bere^7^, Fabian Sebastian Achana^6^, Lucy Owusu1, Dixon Chibanda^4^, Melanie Abas^1^, Benedict Weobong^8,9^, Moses Kumwenda^2^

**1** Institute of Psychiatry, Psychology and Neuroscience, King’s College London, **2** Department of Pathology, Kamuzu University of Health Sciences, **3** School of Public Health, University of Ghana, **4** The Friendship Bench, Zimbabwe **5** Navrongo Health Research Centre, **6** Ghana Health Service, **7** Department of Mental Health, Faculty of Medicine and Health Sciences, University of Zimbabwe, **8** Department of Social and Behavioural Sciences, University of Ghana, Accra, Ghana, **9** Faculty of Health, York University, School of Global Health, Toronto, Ontario, Canada.
