# Supplementary material for: Parental involvement in school-based mental health interventions for young people in low-resource settings: A qualitative study from Zimbabwe and Ghana
Source: PLoS One. 2025 May 9;20(5):e0322954. doi: 10.1371/journal.pone.0322954 (PMC12063816; doi:10.1371/journal.pone.0322954)
Supplement: S1 Checklist — (DOCX) [file pone.0322954.s001.docx]

**Human Participants Research Checklist**

***Complete the following if your study involved human participants or human participants’ data. These questions should be addressed for prospective and retrospective studies.***

1. Did you obtain ethics approval for this study?
   - If yes, please upload (file type “Other”) the original approval document you received from your ethics committee. If the original document is in another language, please also provide an English translation.

___ **Uploaded**

- - If you did not obtain ethical approval, please explain why this was not required below.

1. If you prospectively recruited human participants for the study – for example, you conducted a clinical trial, distributed questionnaires, or obtained tissues, data or samples for the purposes of this study, please report in the Methods:
   1. the day, month and year of the **start and end** of the recruitment period for this study.
   - In Zimbabwe, recruitment was from 20 December 2022 to 30 September 2023. In Ghana,
   1. whether participants provided informed consent, and if so, what type was obtained (for instance, written or verbal, and if verbal, how it was documented and witnessed). If your study included minors, state whether you obtained consent from parents or guardians. If the need for consent was waived by the ethics committee, please include this information.
   - Participants in the study provided written informed consent prior to participation. For minors, parental or guardian consent was obtained alongside assent from the minors themselves. All consent and assent procedures were approved by the relevant ethics review board, ensuring compliance with ethical standards for research involving human participants.

**___ Completed**

1. If you are reporting a retrospective study of medical records or archived samples, please report in the Methods section:
2. the day, month and year when the data were accessed for research purposes
3. whether authors had access to information that could identify individual participants during or after data collection

**___ N/A**
