# Supplementary material for: Parental involvement in school-based mental health interventions for young people in low-resource settings: A qualitative study from Zimbabwe and Ghana
Source: PLoS One. 2025 May 9;20(5):e0322954. doi: 10.1371/journal.pone.0322954 (PMC12063816; doi:10.1371/journal.pone.0322954)
Supplement: Attachment — Submitted filename: Response to reviewers.docx [file pone.0322954.s004.docx]

**Response to Reviewers**

Please include the following items when submitting your revised manuscript:

- A rebuttal letter that responds to each point raised by the academic editor and reviewer(s). You should upload this letter as a separate file labeled 'Response to Reviewers'.
- A marked-up copy of your manuscript that highlights changes made to the original version. You should upload this as a separate file labeled 'Revised Manuscript with Track Changes'.
- An unmarked version of your revised paper without tracked changes. You should upload this as a separate file labeled 'Manuscript'.

**Editor Comments**

1. Please ensure that your manuscript meets PLOS ONE's style requirements, including those for file naming. The PLOS ONE style templates can be found at

<https://journals.plos.org/plosone/s/file?id=wjVg/PLOSOne_formatting_sample_main_body.pdf> and

[https://journals.plos.org/plosone/s/file?id=ba62/PLOSOne_formatting_sample_title_authors_affiliations.pdf](https://eur03.safelinks.protection.outlook.com/?url=https%3A%2F%2Ftrack.editorialmanager.com%2FCL0%2Fhttps%3A%252F%252Fjournals.plos.org%252Fplosone%252Fs%252Ffile%253Fid%3Dba62%252FPLOSOne_formatting_sample_title_authors_affiliations.pdf%2F1%2F010f0195836f0448-8af994f6-8050-41f7-bc6f-f5cda0d245e4-000000%2FtywmOvQJe6JpM6BP_su0925_aBB-P7wJPg7Xm-HAKMw%3D201&data=05%7C02%7Crufaro.mushonga%40kcl.ac.uk%7C5bcc43c6c17146f78d7008dd6054191c%7C8370cf1416f34c16b83c724071654356%7C0%7C0%7C638772636832925294%7CUnknown%7CTWFpbGZsb3d8eyJFbXB0eU1hcGkiOnRydWUsIlYiOiIwLjAuMDAwMCIsIlAiOiJXaW4zMiIsIkFOIjoiTWFpbCIsIldUIjoyfQ%3D%3D%7C0%7C%7C%7C&sdata=i8lrcOE33nARMvgThpTUFD7c3%2B3tWyrWXXMnjK5zbhw%3D&reserved=0).

**Response:** The manuscript has now been formatted as per PLOS ONE’s style requirements

2. Please include a complete copy of PLOS’ questionnaire on inclusivity in global research in your revised manuscript. Our policy for research in this area aims to improve transparency in the reporting of research performed outside of researchers’ own country or community. The policy applies to researchers who have travelled to a different country to conduct research, research with Indigenous populations or their lands, and research on cultural artefacts. The questionnaire can also be requested at the journal’s discretion for any other submissions, even if these conditions are not met.  Please find more information on the policy and a link to download a blank copy of the questionnaire here: <https://journals.plos.org/plosone/s/best-practices-in-research-reporting>. Please upload a completed version of your questionnaire as Supporting Information when you resubmit your manuscript.”

**Response:** A complete copy of PLOS’ questionnaire on inclusivity in global research has been included as supporting information

3. Please provide additional details regarding participant consent. In the ethics statement in the Methods and online submission information, please ensure that you have specified (1) whether consent was informed and (2) what type you obtained (for instance, written or verbal, and if verbal, how it was documented and witnessed). If your study included minors, state whether you obtained consent from parents or guardians. If the need for consent was waived by the ethics committee, please include this information.

**Response:** All additional details requested regarding consent/assent has been provided in the Methods section

4. In the online submission form you indicate that your data is not available for proprietary reasons and have provided a contact point for accessing this data. Please note that your current contact point is a co-author on this manuscript. According to our Data Policy, the contact point must not be an author on the manuscript and must be an institutional contact, ideally not an individual. Please revise your data statement to a non-author institutional point of contact, such as a data access or ethics committee, and send this to us via return email. Please also include contact information for the third party organization, and please include the full citation of where the data can be found.

**Response:** The data underlying the results presented in this study are available from the Institute of Psychiatry, Psychology & Neuroscience, King's College London, United Kingdom (16 De Crespigny Park, London SE5 8AF). The point of contact for the data is Melanie Abas, the Principal Investigator of the project at KCL. As per our ethical approvals, access to the data is restricted to the study team, who are co-authors of this paper. Since participant consent was not obtained for data sharing beyond the study team, we are unable to deposit the data in a public repository or share it with external individuals.

5. Please review your reference list to ensure that it is complete and correct. If you have cited papers that have been retracted, please include the rationale for doing so in the manuscript text, or remove these references and replace them with relevant current references. Any changes to the reference list should be mentioned in the rebuttal letter that accompanies your revised manuscript. If you need to cite a retracted article, indicate the article’s retracted status in the References list and also include a citation and full reference for the retraction notice.

**Response:** Reference list has been reviewed

**Reviewer 1 Comments**

I think it could be helpful to add a sentence highlighting the fact that parental involvement is currently not part of the school based mental health interventions in Zimbabwe and Ghana.

**Response:** The statement requested was added in the introduction section of the manuscript

It would be helpful to explain why the different age groups for the two countries (15-24 in Zimbabwe and 15-18 in Ghana).

**Response:** Additional information on why the different age groups for the two countries (15-24 in Zimbabwe and 15-18 in Ghana) was provided.

Please shed some light on why CMDs are common among young people in Navrongo, Ghana.

**Response:** The requested information was provided

For table 1 consider using sex instead of gender, gender is a social construct that refers to the behaviors and role and identities of people.

**Response:** This was resolved

For table 1 is there a difference between the zeros and the dashes?

**Response:** This was resolved

For the references please check for inconsistencies i.e. some journal names are written in full and others are shortened. References number 5 and 10 (W.H.O and World Health Organization) etc.
**Response:** This was resolved

**Reviewer 2 Comments**

In Methods (Inclusion and Exclusion Criteria), authors mention "active major mental disorder" as an exclusion criterion. Please provide additional details on how that was defined - whether by RA designation, previous diagnosis, use of pharmacotherapy, etc.

**Response:** Additional details requested were provided

In Methods, consider providing additional details that can help contextualize the socioeconomic status of the schools/student participants. For example, if public vs private school. This can be useful since the Discussion mentions logistical barriers to parental involvement.

**Response:** Additional details with regards to socio-economic status of the schools and participants were provided.

In Results/Discussion, consider adding some comparison of responses between the two study sites in Zimbabwe and Ghana. While the responses from both research sites seem to reinforce each other, it would be interesting to mention if there were any differences noted between the sites.

**Response:** Slight comparisons were provided in the results section (PTA meetings) and discussion section as largely the results from both sites reinforce each other.

In Results, the level of subheadings and their formatting needs to be fixed.

**Response:** Resolved
